# The General Medical Council

**Published:** 1908-12-12

**Authors:** 


### The Hospital

A Journal of

The Medical Sciences and Hospital Administration.

New Series. No. 92, Vol. IV. [No. 1164, Vol. XLV.] SATURDAY, DECEMBER 12, 1908.

### THE GENERAL MEDICAL COUNCIL.

Fifty years ago, in pursuance of the Medical Act of 1858, the Home Secretary for the time being summoned to the Hall of the Royal College of Physicians the members of the first General Medical Council. This body was charged by the Act with the duty of creating and maintaining the Medical Register for the information of persons requiring medical aid, the avowed object being the provision of means whereby the public might distinguish between qualified and unqualified practitioners. This year, in commemoration of the Council's Jubilee, its President, Sir Donald MacAlister, who is also Principal and Vice-Chancellor of the University of Glasgow, has been honoured with a K.C.B., a distinction well earned by meritorious service to the profession. The moment is therefore appropriate to a review of the principal achievements of the Council. We have seen that the prime function of this body was the safeguarding of the public. For this purpose a Register of duly qualified practitioners was instituted to guide the public as to the justice or otherwise of claims to medical knowledge; and in order that admission to the Register might be synonymous with proved fitness to practise, the Council was endowed with twofold powers. It was authorised to supervise the admission tests whereby the qualification of practitioners was ascertained, and also to remove from the Register those who, though qualified, had proved themselves unworthy to exercise their rights. In this way the Act of 1858 sought to regulate the admission of practitioners to the ranks of the orthodox profession, and to ensure that these ranks should be capable of purgation in the interests of the public well-being and of the profession's good name. As its President observed in his recent address to the autumn session, the Council began its life with powers and duties which were indicated rather than defined. It had therefore to contend with many difficulties, and, like other of our institutions, to make its way through a tangle of ancient traditions, vested rights, and sacred privileges. Its undefined duties and powers had gradually to evolve their definition at the hand of the Courts, and the nature and extent of its jurisdiction and disciplinary powers had to be declared and delimitated. This evolution could not in the nature of things be other than a slow process, but it has gone on steadily, and is, of course, still advancing. A moment's retrospect will show the immense progress which has resulted from this establishment of a central authority to control and correlate the procedures of licensing bodies. Prior to the Medical Act the Universities of the United Kingdom gave their degrees and the various colleges their diplomas, without any attempt at uniformity of standard in the tests applied to their candidates. Moreover, and perhaps as a necessary sequence, the qualifications thus issued were only locally valid—the degree of a Scottish University affording no title to practise in England, nor that of an English University in Scotland or Ireland. The chaos and inconvenience resulting from such a state of things requires no labouring. Not least among the Council's achievements is the formulation of a standard of qualification, which is accepted throughout the United Kingdom and has given a general validity, as regards these Islands, to the degrees and diplomas of all the authorised licensing bodies.

Another respect in which the institution of a governing body like the Council has done good service is seen in the effect of its disciplinary powers in raising the ethical standard of the profession. We do not believe that the improvement in this particular which has been witnessed by the last half century is to be attributed entirely to the fear of erasure from the Register for conduct "infamous in a professional respect"; undoubtedly many other causes have operated in the advance; nevertheless there is equally no doubt that the penal powers of the Council have exercised a salutary effect in confining to the path of professional virtue those whose notions of integrity permit them a greater freedom than is compatible with the code of honour to which the majority of their colleagues are content to subscribe.

Of the grumbles against the General Medical Council that most commonly heard is concerned with its supposed backwardness in the suppression of quackery. Those who indulge in such complaints are probably not aware how limited are the powers of the Council in this field. It was not established to subdue the quack, but to supply the public with a reputable body of professional men to whom the sick might resort—if they would. The control of the Council does not extend beyond the ranks of the qualified profession and of the institutions which confer qualifications upon its members. Not directly, that is to say; but indirectly much has been done for the suppression of unqualified practice, by means of the embargo laid upon the employment of unqualified assistants by practitioners themselves fully qualified. Prior to the Council's ruling that the employment of unqualified assistants would be considered conduct infamous in a professional respect, it was a common custom for medical men to delegate a considerable portion of their work to such unqualified assistants. The change no doubt operated hardly in the case of poor practices, but it is equally clear that it has contributed to a higher standard of skill in the treatment of the poorer class of patients. As regards the quack, the Council is powerless, execpt in so far as it can initiate legislation. But here again an insuperable obstacle is encountered in public opinion, against which legislation is impossible. Convinced though we may be that the glorification of quackery which we see about us daily is inimical to the best interests of the public, it is idle to disguise the fact that the public dotes on quacks, and that any movement directed against them by the profession would be interpreted as an expression of pure jealousy. There is not a quack nostrum upon the market in support of whose claims the proprietors cannot produce scores of persons quite honestly convinced that they have benefited by the remedy, even though it be proved to consist of nothing but sugar and water. The reverse of the picture seldom becomes prominent, and little is heard outside the profession of lamentable tragedies due to reliance upon charlatans, such as that chronicled last week by the *British Medical Journal* in connection with certain "cancer-curers" of Cardigan. It is useless to blink the fact that so long as human beings remain suggestible, and so long as the race remains liable to chronic organic diseases which admit only of palliation, the irregular vendor of drugs is certain to flourish. And quite apart from the public favour shown to quacks is the material fact that their trade is a vested interest; for the Treasury taxes it, and the press derives no small part of its profits from the advertisements of this irregular brotherhood. Clearly it is unjust to blame the Council for not inviting an almost certain defeat. Whatever its detractors may say, it has done much to bring the standards of professional knowledge and ethics to the level at which they stand to-day, a level which, all things considered, we may claim to be satisfactory.

